# Antidepressant medication during pregnancy and epigenetic changes in umbilical cord blood: a systematic review

**DOI:** 10.1186/s13148-016-0262-x

**Published:** 2016-09-07

**Authors:** Anne-Cathrine F. Viuff, Lars Henning Pedersen, Kasper Kyng, Nicklas Heine Staunstrup, Anders Børglum, Tine Brink Henriksen

**Affiliations:** 1 Perinatal Epidemiology Research Unit, Pediatric Department, Aarhus University Hospital Skejby, Aarhus, Denmark; 2 Department of Obstetrics and Gynecology, Aarhus University Hospital Skejby, Aarhus, Denmark; 3 The Lundbeck Foundation Initiative for Integrative Psychiatric Research, iPSYCH, Risskov, Denmark; 4 Department of Biomedicine and Centre for Integrative Sequencing, iSEQ, University of Aarhus, Aarhus C, 8000 Denmark; 5 Translational Neuropsychiatric Unit, Aarhus University Hospital, Risskov, 8240 Denmark

**Keywords:** DNA methylation, Prenatal exposures, Antidepressants, SSRI, Depression, Review

## Abstract

**Introduction:**

Epigenetic mechanisms are important for the regulation of gene expression and differentiation in the fetus and the newborn child. Symptoms of maternal depression and antidepressant use affects up to 20 % of pregnant women, and may lead to epigenetic changes with life-long impact on child health. The aim of this review is to investigate whether there is an association between exposure to maternal antidepressants during pregnancy and epigenetic changes in the newborn.

**Material and methods:**

Systematic literature searches were performed in MEDLINE and EMBASE combining MeSH terms covering epigenetic changes, use of antidepressant medication, pregnancy and newborns. A keyword search was also performed. We included studies on pregnant women and their children where there was a history of maternal depressed mood or anxiety, a reported use of antidepressant medication, and measurements of epigenetic changes in umbilical cord blood. Studies using genome-wide or candidate-based epigenetic analyses were included. Citations and references from the included articles were investigated to locate further relevant articles. The completeness of reporting as well as the risk of bias and confounding was assessed.

**Results:**

Six studies were included. They all investigated methylation changes. Genome-wide methylation changes were examined in 184 children and methylation status in specific genes was examined in 96 children exposed to antidepressant medication. Three of the studies found an association between use of antidepressant medication during pregnancy and methylation status at various CpG sites measured in cord blood of the newborn. One of these studies found an association in African-Americans, but not Caucasians.

The remaining three studies found associations between maternal mood and epigenetic changes in umbilical cord blood but no association between epigenetic changes and maternal use of antidepressant medication.

**Conclusion:**

The included studies have not established a clear association between use of antidepressant medication during pregnancy and epigenetic changes in the cord blood. Future studies using newer, more wide-ranging epigenetic methods could discover possible new differentially methylated sites. Larger sample sizes and good validity of exposures are warranted in order to adjust for level of maternal depression, other maternal illness, maternal use of other types of medication, and maternal ethnicity.

PROSPERO registration number: CRD42015026575.

## Background

Mood disorders such as anxiety and depression are frequent among women, especially during pregnancy. Up to 20 % of pregnant women may experience symptoms of depression with approximately 7 % of these women experiencing major depression [1, 2]. Relevant treatment of these disorders is important, both to secure maternal and consequently fetal health, but also because maternal depression and anxiety may have adverse effects on child development [3].

In the USA, the prescription rate for antidepressant medication to pregnant women is as high as 8 % [4]. In Denmark, just over 4 % of women redeem at least one prescription for antidepressant medication during pregnancy with a marked increase over the past 10 years [5, 6].

Use of antidepressant medication during pregnancy has been associated with a range of adverse outcomes in the newborn, e.g., congenital heart defects, low birth weight, newborn adaptation syndrome, and persistent pulmonary hypertension in the newborn [7, 8]. Few studies have also investigated the risk of stillbirth and infant mortality, but found no association [9].

Importantly, intrauterine exposure to antidepressant medication might result in long-term adverse effect such as neurodevelopmental delay [10–16] and affected language competence skills [17]. A direct effect of antidepressants on fetal brain development is corroborated by studies on experimental animals in which monoaminergic drugs, such as most antidepressants, produce neurochemical and functional alterations of early brain development [18]. These changes in the monoaminergic system have also been detected in adult animals [18]. One reason for this unsolicited effect could be that monoamines take part in the regulation of brain cell growth and differentiation prior to their functioning as neurotrophic factors [19]. Maternal depression in itself or factors related to this may also play an important role [20].

The biological mechanisms behind these alterations of early fetal brain development after exposure to maternal depression and antidepressant medication are unclear. Epigenetic deregulation may serve as the key through which prenatal exposures could have long-term consequences on the health of the child.

The study of epigenetics is the study of molecular modifications of gene activity that do not change the underlying DNA sequence. The most studied epigenetic phenomenon is DNA methylation at CpG dinucleotides where cytosine nucleotides undergo a controlled process of methylation or demethylation. This alteration potentially leads to a change in chromatin accessibility and protein interaction, thereby inducing gene silencing or increased transcription [21].

The methylation process is important in regulation of cellular differentiation. DNA methylation patterns are inherited through mitotic cell divisions but remain responsive to environmental stimuli, such as stress and pharmacological exposures making them especially vulnerable during embryogenesis [22].

In this review, we examine studies of maternal use of antidepressant medication and DNA methylation patterns in the cord blood of the newborn child.

## Methods

### Search strategy

The electronic databases MEDLINE (http://www.ncbi.nlm.nih.gov/pubmed) and EMBASE (http://www.embase.com) were systematically searched. The strategy was decided after identification of MeSH/Emtree terms and key words not covered by these terms. The searches included different terms for epigenetic modifications, including DNA methylation, histone modification and microRNA, different terms for antidepressant medication, depression, pregnancy, and newborns. No filters were applied, and no language or time restrictions were used. Unpublished studies, abstracts, conference presentations, and lecture notes were excluded. The latest searches were performed on August 14 2015. Titles and abstracts were screened using the selection criteria stated below, and articles were excluded if sufficient information was available in the titles or abstracts to determine that the studies were not eligible. The full text of the remaining studies was read, and the studies were included if found eligible. The electronic database Scopus (http://www.scopus.com) was searched for citations and references for all included articles on August 14 2015. Complete search strategies for MEDLINE and EMBASE are available from the corresponding author (acviuff@clin.au.dk).

### Study selection criteria

The selection criteria were based on PICOS (participants, interventions/exposure, comparison, outcome, and study design) criteria [23].

- Participants: Newborn children with cord blood samples.
- Exposure: Antidepressant medication during fetal life.
- Comparison: The offspring of women who used antidepressant medication to women with no use of antidepressant medication.
- Outcome: Changes in epigenetic patterns in a cord blood samples from the newborn exposed to antidepressant medication compared to the epigenetic patterns of newborns not exposed to antidepressant medication.
- Study design: Original cohort studies were eligible. Editorials, abstracts, comments, reviews and meta-analyses were excluded.

### Data extraction and study assessment

Data from the studies were extracted and added to structured extraction forms. Each of the included studies was assessed for completeness using the STROBE (Strengthening the Reporting of Observational Studies in Epidemiology) Statement [24]. Table 1 gives an overview of the included studies.

**Table 1:** Study overview

Study	Setting/location	*N*	Study period	Exposure measures	Study material	Outcome measures
Oberlander, T. et al. (2008) [22]^c^	Women recruited as part of a study of psychotropic medication use during and following pregnancy. Canada	98 mother-child dyads: - 33 SRI-exposed, depressed - 49 non-medicated (13 depressed, 36 not depressed) - 16 not analyzed	Missing	Depression: - HAM-D - HAM-A - EPDS	- Whole blood from the mother. - Cord blood from the child.	Pyrosequencing: Methylation status of NR3C1 CpG rich region
Soubry, A. et al. (2011) [26]	Newborn Epigenetics Study (NEST), Duke University, Durham NC, USA	436 mother-child dyads: - 44 AD-treated, depressed (SSRI, SNRI, TCAs, SARi, bupropion) - 66 non-medicated depressed - 326 controls	April 2005-June 2008	Depression Antidepressant medication	- Cord blood from the child.	Pyrosequencing: H19 and IGF2 DMR’s of the imprinted Insulin-like Growth Factor 2(IGF2) gene. - 331 IGF2 DMR’s - 345 H19 DMR’s (some analyses contained both iDMRs)
Devlin, A. et al. (2010) [25]^c^	Women recruited as part of a study of psychotropic medication use during and following pregnancy. Canada	98 mother-child dyads: - 33 SRI-exposed, depressed - 49 non-medicated (13 depressed, 36 not depressed) - 16 not analyzed	Missing	Depression - HAM-D - EPDS	- Whole blood from the mother. - Cord blood from the child.	Pyrosequencing: methylation levels of SLC6A4 and BDNF in pregnant women and their infants.
Schroeder, J. et al. (2012) [27]	SCOR or TRC0S^a^ at the WMPH^b^, Atlanta, Georgia, USA	201 mother-child dyads: - 83 currently depressed - 146 with lifetime depression - 151 depressed treated with AD (132 SSRI)	Missing	Lifetime psychiatric illness. (DSM-IV) - SCID - HRSD17 - BDI Antidepressant medication in 2 groups: 1. SSRI, SNRI, TCA 2. Bupropion	- Cord blood from the child.	Methylation array: Illumina Human Methylation27 BeadChip
Non, A. et al. (2014) [28]	Harvard Epigenetic Birth Cohort, Brigham and Women’s hospital, Boston, MA, USA	58 mother-child dyads: - 13 non-medicated depressed. - 22 SSRI treated, depressed - 23 healthy controls	June 2007-June 2009	Depression (no score, reported in the chart) SSRI (4 types)	- Cord blood from the child.	Methylation array and candidate gene analysis: Illumina Human Methylation450 BeadChip: Site-by-site Regional clusters Pyrosequencing Gene ontology
Gurnot, C. et al. (2015) [29]	Women recruited as part of a study of psychotropic medication use during and following pregnancy. Canada	44 women (91 in the cohort): Array analysis: - 12 SRI-exposed depressed - 11 controls (depressed/non-depressed). Pyrosequencing: - 17 SRI-exposed, depressed. - 25 controls (depressed/ non-depressed).	Missing	Maternal mood: - HAM-D Drug levels: - Measured in the maternal blood.	- Cord blood from the child.	Methylation array: Illumina Infinium HumanMethylation27 array. Pyrosequencing: Verification of the methylation status of the CYP2E1 gene (found in array).

*AD* antidepressant medication

^**a**^SCOR (Specialized Center of Research for Sex and Gender Effects) or TRCBS (Translational Research Center for Behavioral Science, tertiary referral center for treatment of perinatal psychiatric illness).^**b**^Emory Women’s Mental Health Program (WMPH). ^**c**^Both studies use the same cohort with different outcome measures

It was not possible to conduct a meta-analysis due to heterogeneity between studies. The PRISMA statement for systematic reviews was adhered to.

## Results

### Study selection

**Fig. 1:**
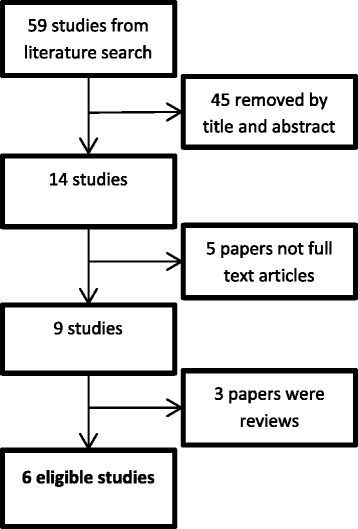
Flowchart showing study selection

After removing duplicates, 59 studies were identified in the original search. These were evaluated by title and abstract leaving 14 studies, and only six of these were original studies (Fig. 1 and Table 1) [22, 25–29]. By using Scopus, we investigated the presence of any useful citations or references. None were found to meet the eligibility criteria. The eligible studies were published from 2008 to 2015.

### Epigenetic changes using an array approach

Three studies investigated methylation patterns using an array-based approach [27–29]. Two studies [27, 29] used the Illumina HumanMethylation27 BeadChip [30] (27K array) and the study by Non et al. [28] used the Illumina HumanMethylation450 BeadChip [31] (450K array) [32]. The 27K array interrogates around 27,000 CpG sites whereas the successor—the 450K array—interrogates more than 480,000 CpG sites.

The study by Schroeder et al. investigated methylation levels in 151 cord blood samples from neonates exposed to antidepressant medication compared to 50 non-exposed samples from neonates using the 27K array. In CpG site cg22464186 located in exon 1 of *TNFRSF21* (tumor necrosis receptor subfamily 21), the methylation was decreased on average by 1.9 %, and in CpG site cg02953306 in exon 1 of *CHRNA2* (cholinergic receptor, nicotinic, α2), the methylation was increased 3 % on average.

The study by Gurnot et al. also used the 27K array and analyzed cord blood from 12 neonates with and 11 without prenatal exposure to selective serotonin reuptake inhibitors (SSRIs). It is difficult to see from the original paper whether the 11 non-medicated controls are depressed or non-depressed. Increased methylation levels were detected at three CpG sites: *CYP2E1* cg13315147 (average difference of 24 %); *EVA1* cg18399703 (average difference of 2 %); and *SLMAP* cg11743795 (average difference of 1 %) (Table 2).

**Table 2:** Results

		Candidate gene studies			Array studies		
Study		Oberlander et al. (2008) [22]	Soubry et al. (2011) [26]	Devlin et al. (2010) [25]	Schroeder et al.^c,d,e^ (2012) [27] (27 K)	Non et al.^b,e^ (2014) [28] (450 K)	Gurnot et al.^c,d,e^ (2015) [29] (27 K)
Gene	CPG sites	*β*-coefficient (*p* value)	*β*-coefficient (*p* value)	Mean methylation % ± SE	Average methylation change % (*p* value)	*β*-coefficient (95 % CI)	*β*-coefficient (SRI-exposed/non-exposed)*
*TNFRSF21*	cg22464186	-	-	-	−1.9 (2.8 x 10^−6^)	NS	NS
*CHRNA2*	cg02953306		-	-	3 (3.1 x 10^−6^)	NS	NS
*CYP2E1*	cg13315147	**-**	-	-	NS	NS	0.30/0.06
*EVA1*	cg18399703	-	-	-	NS	NS	0.06/0.04
*SLMAP*	cg11743795	-	-	-	NS	NS	0.03/0.02
*NR3C1*	CpG1 (D (HAM-D))	0.317 (0.0011)	-	-	-	NS	
	CpG2 (D (HAM-D)) (2nd/3rd trimester)	0.253 (0.066)/ 0.371 (0.003)	-	-	-	-	-
	CpG3 (D (HAM-D))	0.284 (0.023)	-	-	-	-	-
	cg00629244 (D)	-	-	-		−0.006 (−0.01,−0.003)^a^	
*BDNF*	Unspecific	-	–	NS	NS	NS	NS
*IGF2*	IGF2 DMR (D/M)	-	0.72(0.5)/−0.47(0.71)	–	NS	NS	NS
*IGF2*	H19 DMR (D/M) Race interaction (M)	-	0.25 (0.84)/0.54(0.7) 9.18 (0.002)	-	NS	NS	NS
*SLC6A4*	CpG 6 (D)	-	-	4.61 ± 2.1	-	-	-
	CpG 9 (D)	-	-	8.31 ± 3.2	-	-	-
	cg10901968 (M)	-	-	-	NS	−0.007 (−0.01,-0.003)*	NS
*FKBP5*	cg18726036 (D)	-	-	-	NS	0.009 (0.004, 0.01)^a^	NS
	cg07061368 (M)	-	-	-	NS	−0.03 (−0.05,-0.01)^a^	NS
*CRHR1*	cg11731737 (D)	-	-	-	NS	−0.005(−0.01,-0.002)^a^	NS
	Unspecific	-	-	-	NS	NS	NS
*NFKB1*	Unspecific	-	-	-	NS	NS	NS
*NFKB2*	cg23606922 (D/M)	-	-	-	NS	−0.007(−0.01,−0.003)*/−0.01(−0.02,-0.01)*	NS
*DNMT1*	Unspecific	-	-	-	NS	NS	NS
*DNMT3a*	cg15843262 (M)	-	-	-	NS	0.008 (0.004,0.01)*	NS
	*Other CpG sites* ^d^	-	-	-	NS (6 sites of the 42)^d^	42 sites ass. with maternal depression^d^	NS (6 sites of the 42)^d^

The *β*-coefficient represents difference in percent methylation in the exposed vs. the unexposed

*D* Associated with maternal depression, *M* associated with maternal medication, *HAM-D* Hamilton depression scale

*NS* tested and not found statistically significant (FDR-adj. *p* value >0.05). Reported by the specific studies

**-**not tested

*Significantly associated sites (*p* value <0.001).^a^Marginally significant sites.

^b^The Illumina HumanMethylation 450 BeadChip.^c^The Ilumina HumanMethylation 27 BeadChip.

^d^The other significant (FDR-adj. *p* value <0.1) CpG sites consist of 42 sites found with the 450K from a range of other genes not investigated by any of the other studies and only associated to maternal depression but not the maternal use of antidepressant medication. Only 6 out of these 42 CpG sites were also investigated by the other 2 array studies and no association reported

^e^The 27K and 450K have tested several other CpG sites within the listed genes, but not found any statistically significant methylation differences

They validated the *CYP2E1* association in a larger cohort using bisulfite pyrosequencing. Here, they found a high degree of correlation between maternal mood scores and *CYP2E1* DNA methylation values in the newborns exposed to SSRIs, but not in the unexposed newborns suggesting a mood and drug interaction [29]. This means that maternal mood only mattered to DNA methylation with serotonin reuptake inhibitors (SRI) exposure.

The third study using an array approach was the study by Non et al. They used the 450K array to investigate 58 cord blood samples Table 1). Forty-two CpG sites was found in their site-by-site analysis, in which methylation levels were significantly different in those with depressed mothers with no medication compared to the controls. When using a more conservative estimate of significance, they found ten statistically significant sites, e.g., CpG sites cg11846236 (*β* = −0.08, FDR-adj. *p* value 0.031) and cg17913386 (*β* = −0.087, FDR-adj. *p* value 0.046) both located in the first exon of the *COL7A1* gene coding for a part of collagen VII. There were small differences in the degree of methylation in the significant CpG sites ranging from 6 to 9 %. The results contained both increased and decreased levels of DNA methylation. No statistically significant differences in methylation levels in those exposed to SSRIs in pregnancy compared to controls were found.

### Epigenetic changes using a candidate gene approach

Four of the eligible studies used a candidate gene approach [22, 25, 26, 28]. In total, they studied the difference in methylation degree in 11 different candidate genes. Three of the genes were analyzed in more than one of the studies. These studies used bisulfite pyrosequencing. Bisulfite pyrosequencing is considered a robust and reliable method for detection of DNA methylation. The DNA is treated with bisulfite, which converts cytosine residues into uracil, but leave methylated cytosine unaffected, which followed by pyrosequencing yields a highly accurate single nucleotide resolution [32].

#### NR3C1

Two studies investigated the methylation of the human glucocorticoid receptor gene (*NR3C1*) [22, 28]. It is expressed in almost every cell in the human body. The receptor is implicated in both short- and long-term adaptations seen in response to stressors and expression of this gene has been related to hypothalamus-pituitary-adrenal (HPA) stress activity.

Oberlander et al. studied the degree of methylation in the promoter and exon1F of *NR3C1* in both maternal and neonatal blood samples (*n* = 82) and found that children of women with mid-third trimester depressed mood had increased level of DNA methylation at CpG1, CpG2 and CpG3 in exon 1F of *NR3C1* in the umbilical cord blood. They also found that second trimester maternal depressed mood was associated with increased methylation levels at CpG2 in the newborn (Table 2). Methylation status of any of the maternal CpG sites investigated was not associated with maternal depressed mood or with the methylation status in the newborn. They found no association between use of SSRIs and the degree of methylation in the cord blood of the newborn.

Non et al. specifically investigated the degree of methylation in *NR3C1*. They found a marginally increased level of methylation a CpG site cg00629244 in umbilical cord blood associated with non-medicated maternal depression or anxiety (Table 2). This is a novel site in this gene compared to findings from previous studies. Other CpG sites studied by Oberlander et al. were also covered by the micro array in this study, but no significant difference in methylation was found at the sites investigated by both studies.

None of the other studies using the array-based approach found differences in the level of methylation at any CpG sites related to this gene.

#### BDNF

Two studies also investigated the methylation levels in the *BDNF* gene which encodes the brain derived neurotrophic factor. This protein has been linked to increased DNA methylation in suicide victims and suggested as a biomarker in depressive disorders [28].

Non et al. studied this specific gene in the 58 cord blood samples analyzed, and they found no significant change in methylation level at any CpG sites in this gene.

The methylation levels of 12 CpG sites in the *BDNF* gene was in both maternal blood and cord blood studied by Devlin et al. They found that neither maternal nor neonatal promoter methylation status was associated with antenatal mood scores or prenatal SRI exposure.

#### IGF2

Soubry et al. studied two imprinted differentially methylated regions (DMRs) of the *IGF2* gene; the *H19* and *IGF2* iDMRs. The *IGF2* DMR included three CpG sites upstream of exon 3. The *H19* DMR included four sites upstream of the *H19* gene within one of the six CCCTC binding sites. They found no difference in mean methylation percentage when measures from umbilical cord blood were compared between women with antidepressant use and depression during pregnancy. They did, however, find that antidepressant exposure in African-American women was associated with a high methylation profile at the *H19* iDMR corresponding to an increase of 5 % in mean methylation when adjusted for age, smoking, marital status, education, BMI, and birth weight (Table 2).

#### SLC6A4

Two of the studies analyzed *SLC6A4* [25, 28]. *SLC6A4* encodes the serotonin transporter.

In the study by Non et al., one of the ten candidate gene analysis done were on *SLC6A4*. They found that SSRI exposure was associated with a slight decrease in methylation in one CpG site in *SLC6A4*.

Devlin et al. assessed ten CpG sites in the *SLC6A4* gene. The level of methylation in the *SLC6A4* promoter from cord blood of newborns was lower in two CpG sites (CpG 6 and CpG 9) in children of mothers with increased depressed mood symptoms at 26 weeks of gestation. They found no association with SSRI exposure.

None of the statistically significant sites found in either study were the same.

#### FKBP5, CRHR1 and CRHR2, NFKB1 and NFKB2, DNMT1, and DNMT3a

The other seven candidate genes investigated, were all investigated in the study by Non et al. along with *NR3C1*, *BDNF*, and *SLC6A4*. They were chosen for investigation from previous studies [28].

Associations with exposure to non-medicated maternal depression or anxiety were identified at one site in *NFKB2*. This site was also associated with SSRI exposure. CpG sites in the *FKBP5* gene and the CRHR1 gene were marginally statistically associated with non-medicated maternal depression. None of the other sites were statistically associated with neither non-medicated maternal depression nor SSRI exposure (Table 2).

## Discussion

Six studies were included in the review. They had very heterogeneous outcomes with no consistent pattern in their findings.

### Exposure assessment

#### Maternal mood assessment

In the three Canadian studies [22, 25, 29], maternal mood was assessed using The Hamilton Rating Scale for Depression (HAM-D) [33]. For over 40 years, this has been the gold standard in depression diagnostics and grading of severity. The HAM-D has been criticized for being outdated [34], but no other evaluation tool has yet taken its place.

In the studies by Oberlander et al. and Devlin et al., they also used the Edinburgh Postnatal Depression Score (EPDS). It was developed for use postnatally, but later validated for use in the antenatal period [35].

Oberlander et al. also used the Hamilton Rating Scale for Anxiety (HAM-A) prenatally which means, that the women in this study was scored on three different scales for anxiety and depression and the results presented in relation to all three scales and trimester of scoring.

Schroeder et al. used Structured Clinical Interview for Diagnosis (SCID) to assess lifetime diagnosis according to the DSM-IV criteria. They also used the SCID Mood Module to assess major depressive episodes at each visit during pregnancy. The depressive symptoms were assessed using the 17-item Hamilton Rating Scale for Depression (HRSD17) and the Beck Depressive Inventory (BDI) [36]. They used symptoms scores at each visit to assess the area under the curve (AUC) and this was normalized to 40 weeks in order to account for differences in timing of delivery.

In the other two studies, maternal mood was not assessed using a validated rating scale. Neither study had information on duration, onset, or severity.

Non et al. [28] classified patients as having non-medicated depression or anxiety if a note of either of these conditions was made by their obstetricians in their labor and delivery forms, and there was no registration in their medical charts of the use of antidepressant medication.

In the study by Soubry et al., the information on maternal depression was dichotomous (yes/no). Information was obtained from a standardized self- or interviewer completed questionnaire and further verified from medical records.

If there is no information on the level of maternal depression, it is impossible take this into account in the statistical analyses. There may be an association between major maternal depression and methylation changes in the newborn and perhaps also in the adult offspring [37] and performing the analyses without this information could result in overestimation of a true association between the use of antidepressant medication during pregnancy and the methylation changes in the cord blood of the newborn [38].

#### Assessment of maternal use of antidepressant medication

Gurnot et al. did not comment on how they assessed the maternal use of antidepressant medication. It is not obvious how they obtained information on exposure from the participants. They analyzed maternal and neonatal drug levels in maternal blood at delivery and in the umbilical cord blood of the newborn in the exposed group.

Oberlander et al. and Devlin et al. also made no mention of how information on medication exposure status was obtained.

In these three studies only exposure to SSRIs were studied. It is difficult to evaluate the quality of antidepressant exposure due to lack of information.

In the study by Non et al., the information on maternal use of medication was self-reported. They also only included SSRI-exposed women (Zoloft, Prozac, Celexa and Paxil). Dose, treatment window and duration of exposure were not provided.

Schroeder et al. categorized the antidepressants into two groups: SSRIs and bupropion that does not work through serotonergic pathways. The women were evaluated every 4–6 weeks and knowledge of their use of medication was obtained from these visits. The source of information was not specified.

In the last study by Soubry et al., the information on medication use was obtained from the women’s medical records as follows: SSRIs, selective nor-epinephrine reuptake inhibitors (SNRIs), tricyclic antidepressants (TCAs) and serotonin antagonist and reuptake inhibitors (SARIs). Details on duration and dose were not described.

If there is a true association between the use of antidepressant medication and methylation change in the offspring, and some of the women that are actually exposed are classified as unexposed, or the classification is random due to lack of information on medication use, this may bias the estimates of this association towards the null. Thus, for all the studies included, there is a risk of bias due to the lack of detailed information on the use of antidepressant medication, and this may be part of the explanation for their varying outcomes.

### Outcome assessment

#### Microarray-based methods

Three studies used the Illumina HumanMethylation BeadChip—either 27K or 450K array. This way of assessing DNA methylation is cost effective in studies with large sample sizes. Although targeting methylation across the genome, it still covers only a small part of the total methylome, which consists of more than 28 million CpG sites and it is mainly assessing promoter regions. The 450K array covers more than 96 % of known CpG islands and over 99 % of RefSeq genes, and therefore gives wide coverage of the known areas of the methylome [32] and could be preferred if the DMRs of interest lie within these regions. If the aim of a study is to discover DMRs, methylome-wide sequencing-based methods would be optimal [39].

#### Candidate gene approach

Four studies used bisulfite pyrosequencing (Non et al. also used array-based methods) to investigate a candidate gene based on literature mining. Pyrosequencing is an appropriate method for discovering DMRs in candidate gene sequences [32], but it is relatively costly and inefficient when applied genome-wide.

In the epigenetic studies trying to explain later adverse neurodevelopmental deficits in the offspring, it must be taken into account that the biological material on which the arrays were performed originates from the cord blood. The cord blood contains a variety of white blood cell types. Thus, what is found in these arrays of these cells can be the result of other processes, e.g., the inflammatory response from the mother being depressed or taking antidepressant medication. Thus, these differences do not necessarily represent processes in the fetal brain. However, peripheral tissue may still reflect neurological epigenetic phenotype because individual methylations in the brain and blood may be correlated [40].

##### Bias

Only one study (Soubry et al.) reported a response rate (82.3 %) while all the other studies only reported the total number of participants, but not the eligible number. Thus, all studies were potentially prone to selection bias.

Several of the studies obtain the information on the participants by self-report, e.g., in the study by Non et al. they use the self-reported alcohol consumption to exclude women from the study. When obtaining information on behavior like drinking and smoking among pregnant women, it can be feared that they under-report the true use. This problem cannot be rectified seeing that it may be the only way to obtain this information, but it makes the studies prone to information bias and should be taken into consideration.

All the included studies reported statistically significant associations. Thus, publication bias cannot be ruled out, since we have been unable to identify results from unpublished studies.

### Confounding

Confounding was only addressed sparsely in the studies. Soubry et al. chose characteristics associated with depression/antidepressants and methylation with *p* values <0.2. In the study by Non et al., they describe that the covariates they adjusted for were chosen based on results from previous studies.

A list of covariates in the analyses is presented in Table 3. Almost no overlap between the different studies with regard to covariates can be seen.

**Table 3:** Covariates adjusted for in the included studies

Covariates	Oberlander et al. (2008) [22]	Soubry et al. (2011) [26]	Devlin et al. (2010) [25]	Schroeder et al. (2012) [27]	Non et al. (2014) [28]	Gurnot et al. (2015) [29]
Maternal age at delivery		x			x	
Pre-pregnancy BMI		x			x	
Family SES					x	
Neonatal gender				x		
Maternal race		x		x		
Gestational age				x		
Smoking		x				
Marital status		x				
Education		x				
Birth weight		x				
SRI-treatment	x		x			x
Maternal mood			x			x

Many covariates adjusted for fail to fulfill the criteria of being a common cause of both exposure and outcome, hence a confounder [41]. Birth weight is the most frequent covariate included in the analyses. However, birth weight cannot be the cause of maternal depression or use of antidepressants and therefore does not fulfill the criteria for being a confounder [41]. No studies applied causal directed acyclic graphs (DAGs) for covariates adjustment [41] or estimated residual confounding. It could be suggested to investigate the effect of, e.g., maternal illnesses, maternal ethnicity, or additional maternal use of medication.

Exclusion criteria were only reported in three studies. Non et al. and Devlin et al. excluded women using any other type of medication, drinking alcohol during pregnancy, or having any other illnesses. Schroeder et al. excluded women using lithium, stimulants or migraine medication, women with unstable non-psychiatric illnesses requiring pharmacological treatment, or with abnormal thyroid stimulating hormone. These different approaches to covariate adjustment could also be part of the explanation as to why there was a great deal of heterogeneity between the results of the studies.

### Heterogeneity between studies

The genes investigated by the studies using the candidate gene approach were also being investigated by the microarray-based methods (Table 2). None of the studies found similar associations.

This may be due to the lack of complete accordance between CpG sites studied in the various genes. For example, in the studies by Devlin et al. and Non et al., the *SLC6A4* gene was investigated. Only four of the investigated sites were similar in these studies and for these four sites there were similar differences in the degree of methylation. The statistically significant sites found by Devlin et al. were, however, not investigated by the Illumina 450K BeadChip used by Non et al.

The studies did also study many of the same CpG sites without finding similar results. Oberlander et al. investigated the *NR3C1* gene as did Non et al. One of the sites (CpG1) was investigated in both studies. This site had a significantly increased degree of methylation associated with maternal depressive symptoms but was not associated to the use of antidepressant medication in the study by Oberlander et al. No difference in methylation was found in the study by Non et al. at this CpG site.

Also the studies using array-based techniques investigated the same sites without reaching the same results. Gurnot et al. found three CpG sites and Schroeder et al. two CpG sites associated to SSRI exposure using the 27K array. However, they failed to find the same associated CpG sites. In the study by Non et al., they found no CpG site methylation significantly associated with SSRI exposure. It could be expected that the study by Non et al. had found more associated CpG sites when using the 450K array than the studies using the 27K array. This study, however, had a study population of only 58 mother-child dyads, with only 23 exposed to SSRI.

Schroeder et al. studied SSRIs and SNRIs, TCAs, and bupropion in an all combined group. They found two CpG sites associated with the exposure to antidepressant medication. Their result could reflect the effect of one of the other types of medication, and not SSRI. This does, however, not explain why none of the other studies found any CpG sites associated with SSRI exposure. The cause of this discrepancy is not evident but may be due to differences in exposure assessment. The timing of exposure during pregnancy may be important. This information is not available from any of the studies.

Several other external factors may modify the methylation status apart from maternal mood and treatment with antidepressant medication. Maternal smoking during pregnancy has been associated with the degree of methylation in cord blood samples in several studies [42–44]. One of these studies found that maternal smoking was inversely associated with *IGF2* methylation, which could be one of the mechanisms through which fetal growth is affected by maternal smoking during pregnancy. Race has also been associated with differential methylation in newborns [45].

Maternal use of antiepileptic drugs (AED) over a longer period of time has been associated with a decrease in cord blood DNA methylation [46]. Both an average global decrease and significant decrease in specific genes was found associated with the duration of prenatal AED exposure. Perfluorinated alkyl compounds (PFOA) have been inversely associated with global DNA methylation [43]. Previous studies have also investigated the methylation pattern in cord blood of newborns in relation to sex and gestational age [47, 48]. These studies indicated that gender and gestational age may play a role in the level of methylation.

The methylation differences found in the studies were all small, and some of the authors doubted whether their results were biologically significant in spite of them being statistically significant. However, even small differences in methylation may have consequences for the transcription [49]. Therefore, it is very difficult to state whether even a very small difference in methylation is biologically relevant and further studies are needed to answer this question.

### Interpretation

Many of the reported statistically significant sites in the studies are of biological interest. Schroeder et al. found two CpG sites associated with the use of antidepressant medication. *TNFRSF21* is also known as death receptor 6 (DR6) and plays a role in apoptosis in both developing neurons and developing lymphocytes. *CHRNA2* is a subunit of nicotinic acetylcholine receptors and located in a region of chromosome 8p that is suggested to contribute to psychiatric and neurodegenerative disorders [27]. Gurnot et al. found a statistically significant CpG site within the *CYP2E1* gene. *CYP2E1* has previously been associated with psychogenic stressors and one hypothesis is that CYP2E1 functions as a “buffer” against the adverse effects of SSRI exposure [29]. Soubry et al. studied the methylation of the imprinted *IGF2.* The *IGF2* gene plays an important role in embryonic and fetal growth. Prenatal exposure to adverse events has resulted in altered methylation which could lead to deregulated expression of the IGF2 gene [26].

Out of the candidate genes studied by Non et al. one site in *NFKB2* was significantly associated to both non-medicated maternal depression and SSRI exposure. This gene is involved in the transcription regulation of pro-inflammatory cytokines and has been associated with psychosocial stress [28].

Also, marginally statistical significant sites were discovered; e.g., one CpG site in the *FKBP5 gene.* This is an important regulator of the glucocorticoid receptor complex. Demethylation in this region has been associated with trauma during childhood [28].

The study of epigenetics may offer important knowledge when it in time could be desired to treat a certain exposure related disease by epigenetic modulation or use the epigenetic change as a biomarker in detecting individuals at risk of adverse outcomes following certain external exposures.

## Conclusion

From reviewing the literature, it is still uncertain whether the use of antidepressant medication is associated with epigenetic changes in the newborn child. In light of the increasing use of antidepressant medication by pregnant women worldwide, further studies are warranted. In future studies, it would be of great interest to use a whole genome based sequencing approach when investigating the methylome of the newborn children. This approach would yield information from other genomic regions than the ones investigated so far and enabling more adequate epidemiological use of methods when adjusting for confounders that might influence the epigenetic landscape.

## Abbreviations

DMR: Differentially methylated regions
SARIs: Serotonin antagonist and reuptake inhibitors
SNRIs: Selective nor-epinephrine reuptake inhibitors
SSRIs: Selective serotonin reuptake inhibitors
TCAs: Tricyclic antidepressants
